# Pathways to increase the dissymmetry in the interaction of chiral light and chiral molecules†

**DOI:** 10.1039/d1sc02335g

**Published:** 2021-05-21

**Authors:** Jake L. Greenfield, Jessica Wade, Jochen R. Brandt, Xingyuan Shi, Thomas J. Penfold, Matthew J. Fuchter

**Affiliations:** Department of Chemistry and Molecular Sciences Research Hub, Imperial College London, White City Campus 82 Wood Lane London W12 0BZ UK m.fuchter@imperial.ac.uk; Department of Materials, Imperial College London Exhibition Road SW7 2AZ UK; Centre for Processable Electronics, Imperial College London, South Kensington Campus London SW7 2AZ UK; Chemistry – School of Natural and Environmental Sciences, Newcastle University Newcastle upon Tyne NE1 7RU UK

## Abstract

The dissymmetric interaction between circularly polarised (CP) light and chiral molecules is central to a range of areas, from spectroscopy and imaging to next-generation photonic devices. However, the selectivity in absorption or emission of left-handed *versus* right-handed CP light is low for many molecular systems. In this perspective, we assess the magnitude of the measured chiroptical response for a variety of chiral systems, ranging from small molecules to large supramolecular assemblies, and highlight the challenges towards enhancing chiroptical activity. We explain the origins of low CP dissymmetry and showcase recent examples in which molecular design, and the modification of light itself, enable larger responses. Our discussion spans spatial extension of the chiral chromophore, manipulation of transition dipole moments, exploitation of forbidden transitions and creation of macroscopic chiral structures; all of which can increase the dissymmetry. Whilst the specific strategy taken to enhance the dissymmetric interaction will depend on the application of interest, these approaches offer hope for the development and advancement of all research fields that involve interactions of chiral molecules and light.

## Abstract 2

This perspective explores the dissymmetric interaction between circularly polarised (CP) light and chiral molecules. Such interactions are central to many applications from next generation displays to asymmetric photochemical synthesis.

## Graphical abstract

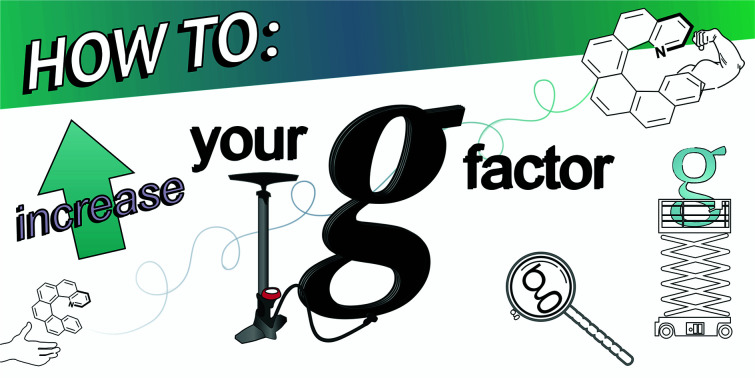

## Introduction

**Fig. 1:**
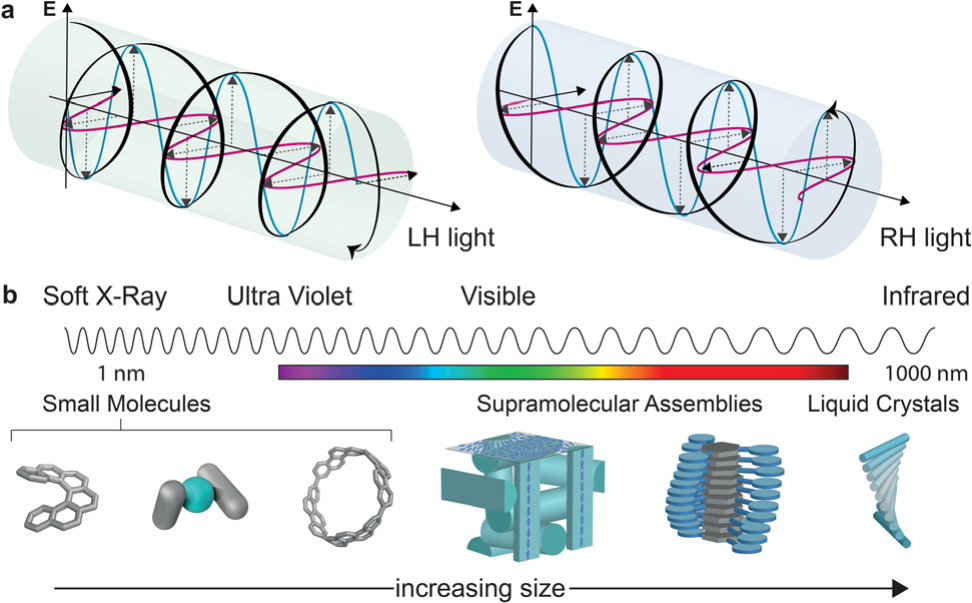
(a) Schematic representation of electromagnetic (EM) waves yielding CP light, (b) examples of various sized systems arranged in order of increasing size and their relation to the wavelength of light in the UV-vis region of the EM spectrum.

Considerations of chirality are embedded into the very fabric of chemistry. The ability to synthesise one enantiomer of a chiral substance over another continues to provide an excellent intellectual challenge and provides access to high value products such as pharmaceuticals. Of course, molecules are not the only objects that can be chiral. Instead, chirality is a property of symmetry and shape that manifests across multiple length scales and throughout the natural world. Beyond molecular chirality, one important example with respect to this perspective is the chirality of light, best exemplified by circularly polarised (CP) light. CP light can be described as two plane polarised waves of equal amplitude, at right angles to one another, but with a quadrature phase relationship. The resultant summed electric field vector is of constant magnitude and rotates at a fixed rate, tracing a helix as the wave travels through space (Fig. 1a). Given that a helix is a chiral object, CP light is chiral light, with two “enantiomeric” left-handed (LH) and right-handed (RH) mirror image forms. The interaction of CP light with chiral molecules (and *vice versa*) underpins a range of spectroscopies already routinely used to interrogate chiral substrates – (electronic/vibrational) circular dichroism (ECD, VCD), optical rotation/rotatory dispersion (ORD), Raman optical activity (ROA) – and continues to be of interest in cutting edge developments in the measurement sciences.^1–4^

This perspective considers the nature of the relationship between chiral molecules and CP light, but from a more preparative standpoint: the selectivity of CP light absorption by chiral molecules, or the use of chiral molecules to selectively produce CP light of a preferred handedness.

The aim to use CP light in asymmetric synthesis is a historically important example. As early as the 19^th^ century, it was suggested that CP light could be used for the selective production of an enantioenriched substance, and CP light-dependent photochemical reactions remain a candidate for the origin of the homochirality of life.^5,6^ But, despite several elegant studies, including varying the photon energy and dual wavelength approaches,^7,8^ CP photochemistry has not become a mainstream technique in asymmetric synthesis. The reason for this is very simple: the enantioselectivity in such reactions is predominately governed by the absorption selectivity of CP light by the enantiomeric starting materials (*i.e.* the ECD of a substance).^9^ Such selectivity is quantified by the so-called dissymmetry or *g*-factor (described in more detail later). While |*g*| = 2 expresses total selectivity for CP light, the *g*-factor tends to be very low (<10^−2^) for electronic transitions important for organic photochemistry. It follows that low differential selectivity in the absorption of CP light between enantiomeric substances equals low stereoselectivity in the resultant photochemistry.

If CP light is generally not very effective at producing chiral molecules with high enantioselectivity, how good are enantiopure emissive molecules at generating CP light of a preferred handedness? The emission of CP light is central to the operation of several next-generation photonic devices, including enantioselective biosensors, efficient displays and security inks.^10,11^ However, if one considers emissive small organic molecules as a representative example, real-world applications are (similar to asymmetric synthesis) limited by the low dissymmetry linked to the electronic transition. A comparative study has previously shown that the emission dissymmetry of CP light from a chiral emissive small molecule is often linearly proportional to, and smaller than, the absorption dissymmetry.^12^ Thus, if the selectivity in the absorption of CP light is low, the emission will be comparably so. This outcome has resulted in a broadly accepted “molecular” dissymmetry factor for the emission from enantiopure small molecules, where the light generated usually displays a dissymmetry of <10^−2^.^13^

While we describe key fundamental aspects in much more detail below, one ‘rule of thumb’ that has been used to explain the disappointing selectivity in these outcomes is molecular size.^14^ Many small organic molecules are shorter than 1 nm at their widest point and are therefore considerably smaller than the hundreds of nm wavelengths of CP light at relevant energies (Fig. 1b). Therefore, small chiral molecules do not ‘feel’ a significant degree of the twist of the light (and *vice versa*). But is this size mismatch universal for chiral light–matter interactions and is it possible to further boost absorption or emission dissymmetry beyond the “molecular” limit?

In this perspective, we present some of the fundamental mechanisms at play in the absorption and emission of CP light by chiral systems and explore how changes in the molecular structure can be leveraged to increase the dissymmetry of CP light–matter interactions. With such a vast array of potential molecules, materials and mechanisms, we do not aim to be exhaustive in our analysis. In particular, the mechanisms that underpin the interaction of light with chiral metamaterials, perovskites and Thermally Activated Delayed Fluorescence (TADF) molecules are complex and beyond the scope of this perspective. Nonetheless, we hope that the representative examples we have selected not only explain the current state-of-the-art, but signpost opportunities for future research directions.

## Intrinsic chiroptical activity: the case of the chiral chromophore

The differential absorption or emission of CP light can be quantified by the so-called dissymmetry or *g*-factor (eqn (1)).1
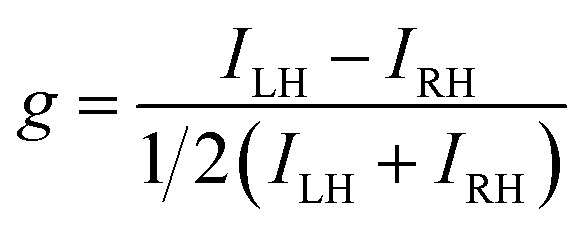
Here, *I*_LH_ and *I*_RH_ refer to the left-handed and right-handed absorption/emission intensities, respectively.

For small molecules and systems that lack long-range chiral order, CP emission and absorption depends on the chirality of the ground and excited electronic states, *i.e.* on the chirality of the chromophore. Here we use the phrase ‘intrinsic chiroptical activity’ because the absorption or emission of CP light is intrinsic to the (isolated) chromophore itself. Other nomenclature can be found in the literature, including ‘natural optical activity’;^15^ although the use of this term has been criticised.^16^

In such instances, the dissymmetry of absorption of an electronic transition between the *i* and *j* states can be defined by the following expressions:^12^*R* = Im***μ***_*ij*_·***m***_*ij*_*D* = |***μ***_*ij*_|^2^ + |***m***_*ij*_|^2^2
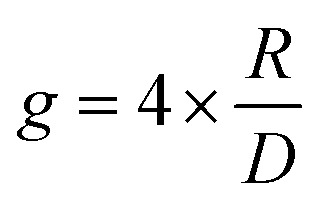
Within the limit of |***μ***_*ij*_| ≫ |***m***_*ij*_|, which is usually the case of the molecular scales, the dissymmetry can be expressed as:3
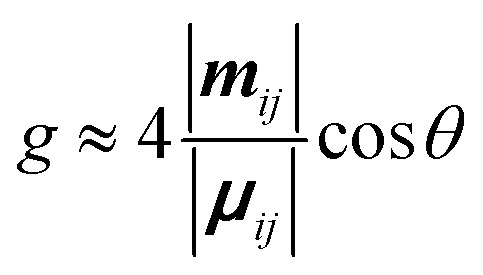
Here, *R* refers to the rotational strength, *D* refers to the dipole strength, ***m*** refers to the magnetic dipole moment and ***μ*** the electric transition dipole moment. *θ* is the angle between the transition dipole moments.

Eqn (3) can also be used to calculate the dissymmetry of emission (*g*_lum_). Whilst *g*_abs_ quantifies the dissymmetry of the thermally equilibrated electronic ground state, *g*_lum_ reflects the dissymmetric structure of the emissive excited state.

As can be seen from eqn (3), the dissymmetry is sensitive to both the magnitude and relative orientations of the magnetic and electric transition dipoles within the chromophore. In most small molecules, at least when considering *dipole-allowed* electronic transitions, the magnetic transition dipole moment (***m***) is overwhelmed by the electric transition dipole moment (***μ***), and their non-optimised alignment (*θ*) results in very small *g*-factors: the ‘molecular’ *g*-factor. This seemingly unescapable physical limit is obvious from the chiral small molecule literature, where the vast majority of molecules exhibit *g*-factors <10^−2^.^12,13^ The validity of this trend is born out of the electric dipole approximation, which assumes the wavelength of light is much larger than the typical size of a molecule.^17^ Consequently, it is apparent that molecular size is critical when considering the intrinsic chiroptical activity of a structure. Indeed, this size mismatch effect is not limited to the absorption or emission of electromagnetic radiation in the UV and visible (UV-Vis) range. The detection of CD signals for vibrational transitions (vibrational circular dichroism, VCD) is a powerful technique that can often allow the determination of the absolute configuration of a chiral molecule in solution. As the wavelengths associated with vibrational transitions are about ten times longer than for the electronic transitions of CD in the UV/vis range (electronic CD, ECD), typical VCD *g*-factors are smaller than those observed in ECD and remain on the order of 10^−4^.^18^

Significantly enhancing chiroptical activity requires a breakdown of the electric dipole approximation, which indicates that *g*-factors that exceed the ‘molecular’ limit can be achieved either by decreasing the wavelength of light^19^ or by exploiting larger molecules and structures. But is there evidence for this? Well, there are certainly cases where an increase in the size can improve the *g*-factor of molecular systems.

**Fig. 2:**
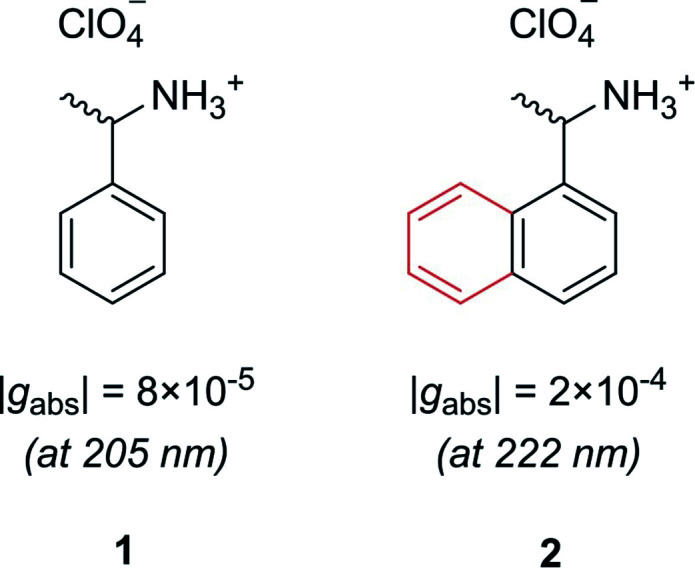
Example showing how increasing the molecular size, in this case a phenyl ring to a naphthyl ring, can increase the dissymmetry of a small molecule.^20^

Let us first consider molecules consisting of a simple aromatic chromophore with an adjacent chiral centre. In such a scenario, the chirality of the molecule is not heavily coupled to the chromophore. This leads to a weak chiroptical response, *i.e.* the chromophore exhibits a low degree of asymmetry. For example, phenylethylammonium perchlorate, **1**, exhibits a very low dissymmetry value of |*g*_abs_| = 8 × 10^−5^ at 205 nm (Fig. 2). Despite the low intrinsic chiroptical activity of **1**, the *g*-factor can be increased by extending the size of the chromophore to a naphthyl ring, **2**, which more than doubles the dissymmetry, |*g*_abs_| = 2 × 10^−4^ at 222 nm.^20^

Extending the size of a chromophore is not limited to conventional covalent chemistry; supramolecular and dynamic-covalent assembly have proved to be particularly important strategies: using point chirality as a means to bias the assembled system into a helical structure of one dominant handedness.^21,22^ In such situations, the assembly adopts a helical configuration, resulting in a chiral environment at the chromophores, which dominates the chiroptical response.

**Fig. 3:**
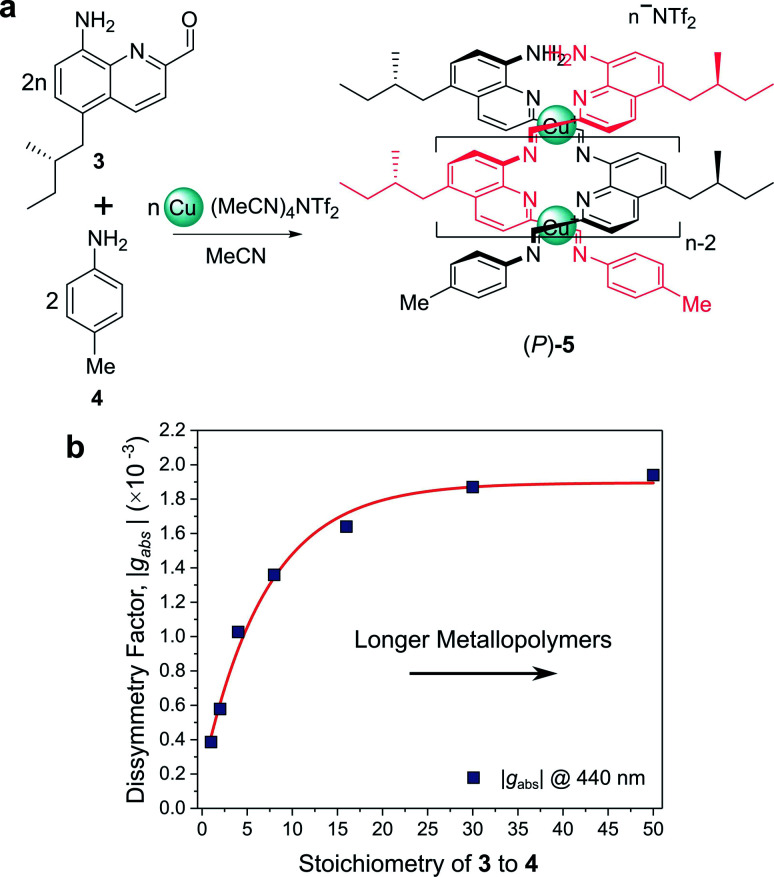
(a) Chiral monomer **3** assembles into double helical metallopolymer **5**, in the presence of Cu^+^ ions. (b) The |*g*_abs_| of **5** increased as a function of metallopolymer length (controlled by varying stoichiometry of **3** and **4**), plateauing at *ca.* 15 repeat units. This figure has been generated from the data in the ref. ^23^.

For example, Nitschke and co-workers have demonstrated how the length of self-assembled helical metallopolymers impacts their *g*-factors (Fig. 3a).^23,24^ In the disassembled state, the free enantiopure monomers are CD silent due to the remoteness of the chiral centre from the quinoline-based chromophore.^23^ Upon self-assembly, the quinoline motifs coordinate to Cu^I^ ions, forming a double helix comprised of conjugated strands, and exhibits bands in the CD spectrum. The |*g*_abs_| of both the π–π* and metal-to-ligand charge-transfer absorption bands were shown to increase as a function of metallopolymer length from approx. 0.4 × 10^−3^ to 1.9 × 10^−3^ (Fig. 3b). This length dependence, paired with a red-shift in the UV-vis and CD absorption bands, can be attributed to an increase in exciton delocalisation along the helically wrapped backbone. The plateau after approximately 15 repeat units (Fig. 3b) can be attributed to increased disorder within the structure.^23,24,26^ While this example broadly supports the concept that increasing molecular size increases chiroptical activity, it is evident that the degree of helical asymmetry, coupling between nearby chromophores and long range structural order are also important.

**Fig. 4:**
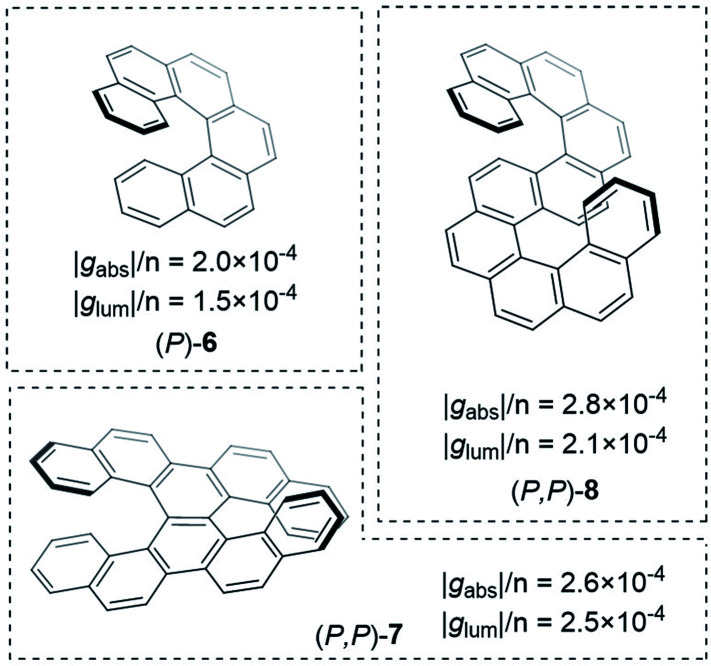
Examples of helicenes displaying different dissymmetry factors normalised to the number of benzene rings in the structure.^30^

Improving the coupling between the element(s) of chirality and the chromophore is an important handle to improving the dissymmetry beyond the examples shown thus far. While there are now numerous designs for improved small molecule chiral chromophores, perhaps the most famous examples are the helical aromatics known as the helicenes, **6** (Fig. 4).^13^ In dilute solution, small helicenes demonstrate |*g*_abs_| values <10^−2^.^12^ As the chromophores of helicenes are embedded into a helical framework, they provide an interesting platform to evaluate the impact of molecular size on their intrinsic chiroptical response.

An obvious way to increase the size of a helicene is to extend the molecule along its helical axis through the addition of fused aromatic rings.§§Although there is currently significant interest to laterally extend helicenes into so-called twisted nanographenes.^117^ The outcome of this procedure has been systematically studied by Mori, Inoue and co-workers, comparing predicted chiroptical responses to experimental data.^27^ They found that when considering the transition that runs perpendicular to the helicene *C*_2_ axis, |*g*_abs_| linearly increases with the number of aromatic rings. Interestingly, the linear trend was found to be discontinuous after 6 rings (hexahelicene, **6**, Fig. 4), which correlates to the length at which the molecule achieves one full helical turn. Extrapolation of this trend to a potentially infinite helicene, while considering a maximal effective conjugation length of [*n*]helicene at *n* ≈ 50, led to a predicted maximal |*g*_abs_| of ∼0.09 for [50]helicene.

Further experimental study of longer helicenes is hampered by the synthetic challenge of making such molecules. Nonetheless, exciting examples of long helicenes are starting to emerge, and some of these show very large chiroptical responses.^28^ A pure assessment of size *versus* chiroptical response is neither trivial nor appropriate in many of these cases, as other mechanisms such as chromophore coupling (discussed in more detail below) clearly play a role.^29^

Building on the rich history of helicenes as molecules with high chiroptical activity, Mori and co-workers computationally investigated how two carbohelicene scaffolds in close proximity can influence the *g*_-_factor.^30^ When arranging hexahelicene **6** as dimers, the average |*g*_lum_| increased compared to the isolated monomer, **6**; the |*g*_lum_| value of the dimer varied depending on the relative orientation of the two monomers. Based on these theoretical studies, the team synthesised and measured double hexahelicene structures with an X- and S-orientation, aiming to mimic dimeric assemblies in a conjugated covalent framework (Fig. 4). To deconvolute the impact of orientation and increased size, the authors normalised the dissymmetry factor to the number of benzene units in the structure. Based on this measure, the X-shaped double helicene, **7**, showed |*g*_lum_| and |*g*_abs_| values 1.7 and 1.3 fold higher (per benzene ring) than **6**. S-shaped helicene **8** displayed 1.4 (|*g*_lum_| & |*g*_abs_|) higher values per ring.

Of course, if one wanted to further inspect the impact of size effects in even larger molecules, it would make sense to consider polymeric systems. Such systems can give rise to very large dissymmetry (|*g*_abs_| >1), in part due to the increased delocalisation of their chromophore.^31^ It should be emphasised that the large chiroptical responses of polymeric systems tend to occur in the condensed phases, *i.e.*, in the absence of aggregation or chromophore coupling the dissymmetry is very small; polymeric systems are revisited later in this perspective.

In summary, it is evident that the relationship between the dimensions of light and the dimensions of molecular systems influences the magnitude of the chiroptical response. It is apparent that increases in the size of the chiral chromophore *can* lead to increases in the dissymmetry. However, within a given molecular framework, the impact of increasing chromophore size on the dissymmetry may be modest, and it is difficult to cross-compare very different structures. This is, in part, because the identity of the chirality element(s), the asymmetry of the chromophore, and supramolecular order all play a role in determining the chiroptical response. Therefore, while it appears that molecular size is important, other aspects of the system in question should also be considered.

Perhaps the most important observation thus far is that the majority of chiral chromophores struggle to break through the ‘molecular’ limit of *g* < 10^−2^. From a technological standpoint this is clearly problematic, where large chiroptical responses (*i.e.* the strong absorption/emission of CP light) are essential.^10^ The remainder of this perspective presents alternative approaches to enhance the chiroptical response, from rational molecular design, exploitation of forbidden transitions, to considerations of the properties and structure of both the chiral molecules and light itself.

## Enhancing intrinsic chiroptical activity beyond the ‘molecular’ limit through design

Given the understanding that underpins eqn (3), it is logical that adjusting the relative strengths and orientations of the magnetic and electric transition dipole moments provides a strategy to enhance the magnitude of the dissymmetry.^12^ This section explores two key examples of this strategy.

### Exploiting forbidden transitions

The strength of an electronic transition is determined by the magnitude of the electric transition dipole moment (***μ***), which can be assessed using a set of selection rules. For an electronic transition to be allowed, it must conserve spin (spin selection rule) and involve a change in parity of the wavefunctions. In contrast, for an allowed magnetic dipole transition (*i.e.* non-zero |***m***|), the parity of the initial and final state of the transition must be conserved. From a different perspective, the electric transition dipole moment involves the translation of charge density, while the magnetic transition dipole requires a rotation of charge density. This combination (translations and rotation) is responsible for creating the helical interaction of the light. The difference in the selection rules means magnetically allowed transitions are usually weakly emissive. In such forbidden transitions, the increased |***m***|/|***μ***| can amplify the dissymmetry (eqn (3)) but often to the detriment of the overall emission intensity.^32,33^

**Fig. 5:**
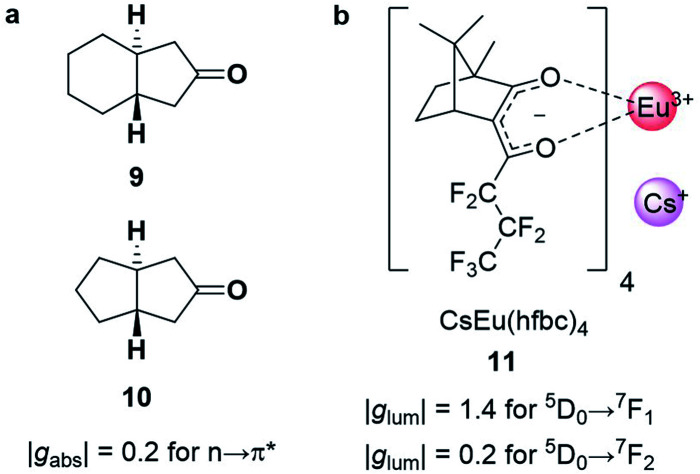
(a) Cyclic ketones, **9** and **10**, with strong |*g*_abs_| of 0.2 for the n→π* transition.^34^ (b) The structure of the Cs[Eu(hfbc)_4_] complex which possesses the highest solution |*g*_lum_| for a small molecular entity (1.4).^37^

Chiral cyclic ketones such as **9** and **10** (Fig. 5a), are an important class of chiroptically active molecules; studies into the emission of CPL by small organic molecules were limited to this substrate class for approximately three decades.^12^ These molecules show strong |*g*_abs_| values of up to 0.2 due to their ***μ***-forbidden but ***m***-allowed *n*→π* transitions, which dominate the chiroptical response (eqn (3)).^34^ Such high |*g*_abs_| allowed for seminal work in the asymmetric synthesis of chiral molecules using CPL photochemistry.^35^ Unfortunately, most ketones are not suitable for CPL emissive applications due to their low luminescence quantum yields (***μ***-forbidden) and the limited tuneability of their emission wavelengths. Furthermore, their |*g*_lum_| values are often considerably lower than |*g*_abs_| (0.03 for the ketones in Fig. 5a).^12^ This discrepancy in the *g*-factor of absorption and emission is a consequence of geometric changes of the C=O bond in the excited state.^12,36^

The strongest |*g*_lum_| values for small molecular entities in dilute solution have been recorded for lanthanide complexes. As the f→f transitions of lanthanides are Laporte-forbidden, direct absorption by a lanthanide ion is impractically weak. Yet high overall quantum yields can be achieved for lanthanide complexes by employing ligands as *antennas*: these ligands absorb light of shorter wavelengths and transfer the energy to the lanthanide, which can then radiatively relax.^38^ A record solution |*g*_lum_| of 1.4 was achieved for the ^5^D_0_→^7^F_1_ transition with Cs[Eu((+)-hfbc)_4_], **11**, (Fig. 5b), with a still respectable |*g*_lum_| of 0.2 for the ^5^D_0_→^7^F_2_ transition.^37,39^ However, the quantum yield of complex **11** is only 3%, illustrating the need to balance both brightness and the emission dissymmetry.^32,33^ Such highly dissymmetric emissions were used to produce phosphorescent OLEDs that emit CP light^40,41^ and could be of interest for imaging in a biological context,^42^ for high information density molecular barcodes or use in security tags.^11,43^

**Fig. 6:**
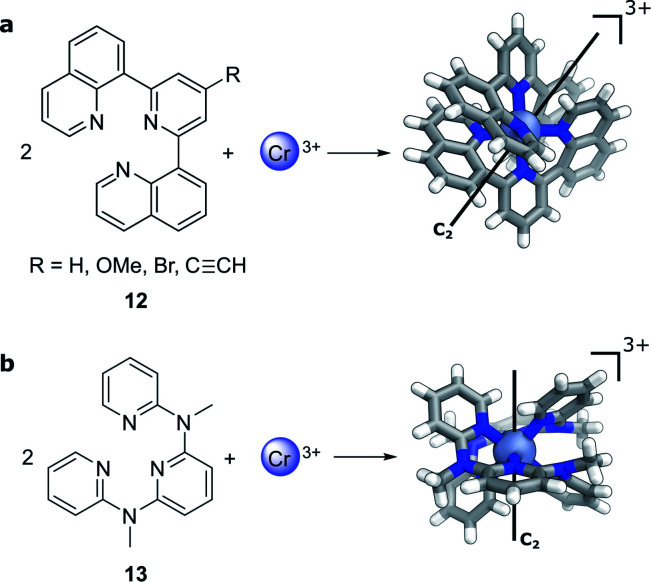
The crystal structure of two Cr(iii) complexes displaying |*g*_lum_| values of (a) 0.2 (R = H, ref. 44; R ≠ H, ref. 46) and (b) 0.093 (ref. 45).

Compared to lanthanide f→f transitions, transition metal d→d transitions typically show a lower dissymmetry. This is due to the apparent breakdown of the Laporte selection rule by vibronic coupling of the electronic excitation, thus leading to larger values of ***μ***. Yet Cr(iii) complexes^44–46^ offer hope towards using earth-abundant metal systems to achieve high dissymmetry values combined with high luminescence quantum yields. Notably, photoresolution of racemic chiral octahedral Cr(iii) complexes is another historically important example of asymmetric synthesis using CPL.^35,47^ In such complexes, two achiral, tridentate ligands helically wrap around a chromium ion to form a twisted, pseudo-octahedral complex. In solution, the maximum |*g*_lum_| factors are 0.2 with 5.2% total luminescence quantum yield for **12** (R = H, Fig. 6a)^44^ and |*g*_lum_| = 0.093 for **13** (Fig. 6b)^45^ with up to 30% luminescence quantum yield for a deuterated derivative. In 2021, one of the research teams further demonstrated that through judicious modification of the ligand a high brightness of CPL emission (*B*_CPL_)^32,33^ can be achieved, reaching 170 M^−1^ cm^−1^ for |*g*_lum_| of 0.2 (R = C≡CH) and a quantum yield of 17% at *ca.* 750 nm (R = OMe).^46^ The highly dissymmetric emissions are achieved for the phosphorescent ^2^E→^4^A_2_ and ^2^T_1_→^4^A_2_ transitions in the NIR region that are analogous to the R1- and R2-ruby laser lines.

### Engineering the transition dipole moments

While exploiting forbidden transitions allows for high *g*-factors due to an increased contribution of ***m***, this strategy most often comes at a cost of lowering the absorption strength and/or luminescence quantum yield. An alternative approach is to engineer the structure of the molecule to better balance the contributions of |***μ***| and |***m***| and to optimise the angle between them (eqn (3)).

**Fig. 7:**
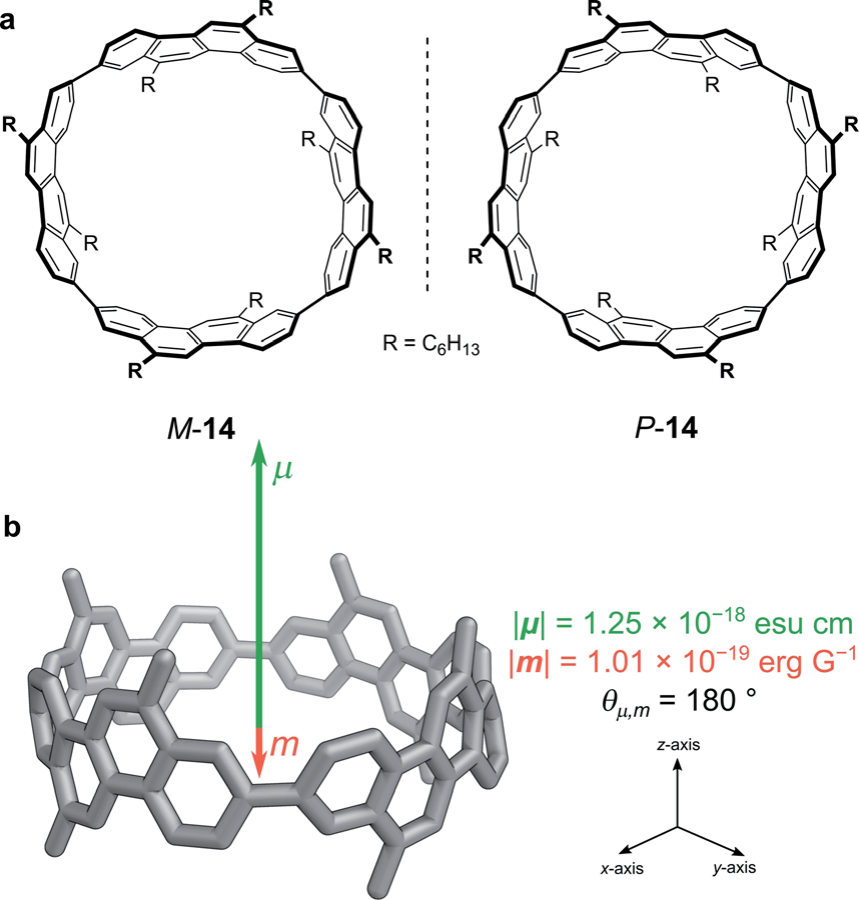
(a) The two enantiomers of (12,8)-[4]CC. (b) The orientation of the ***μ*** and ***m*** transition dipole moments of (P)-(12,8)-[4]CC.^48^

One elegant means to achieve this for π→π* transitions was demonstrated by Sato, Isobe and co-workers using a cylindrical molecule, **14** (Fig. 7). In this example, the chromophore extends over the entire cylindrical molecule, such that the sum of the ***μ****’*s of individual electrons cancel out in the xy-plane (***μ***_*x*_ = ***μ***_*y*_ = 0, ***μ***_*z*_ ≠ 0). In general, the ***m*** originates from the change in angular momentum due to the movement of an individual electron during an electronic transition and is proportional to the vector product of the displacement vector ***r*** and ***μ***, (***r*** × ***μ***). Since ***μ***_*x*_ = ***μ***_*y*_ = 0, and ***r*** radiates towards the outside of the cylinder, the overall ***m*** is orientated antiparallel to the *z*-direction. The resulting ***μ*** is parallel to the cylinder axis and at *θ* = 180 to ***m*** (Fig. 7b). As cos *θ* is −1 at 180°, the dissymmetry is impressively large (eqn (3)), with a solution |*g*_lum_| of 0.15 at 443 nm and a quantum yield of 80%.^48^

In 2021, Matsuda and co-workers demonstrated an alternative approach to engineer the transition dipoles for helicenes,^49^ where they introduced electron donating and withdrawing substituents into a [7]helicene framework in order to tune the frontier molecular orbitals. Judicious choice of the identity and placement of substituent groups allowed for an increase in |***m***|, while also rendering the transition partially symmetry-allowed. Notably, the S_1_ state of unsubstituted [7]helicene displayed a low |***m***|, whilst a [7]helicene substituted with a combination of electron donating and withdrawing groups afforded a 52-fold greater value of |***m***|. This resulted in amplified *g*-factors and quantum yields. The authors noted that |***m***| tends to be larger when there is a change in the bonding and antibonding character of highly overlapped HOMO and LUMO molecular orbitals, suggesting that further design rules to rationally engineer larger values of |***m***| are starting to emerge.

In summary, the use of forbidden transitions and careful design of the molecular structure can maximise |***m***|, |***μ***| and cos *θ*, which allows one to access *g*-factors larger than would ordinarily be expected for small molecules. This strategy is not without its challenges and limitations however, including: low absorption/emission strengths, synthetically challenging molecules and the need to balance transition dipole engineering with application-specific structural motifs—for example, chromophores to achieve a particular absorption/emission colour. Nonetheless, tailored design, supported by computation is clearly a strategy that can be adopted for boosting dissymmetry.

## Enhancing chiroptical activity through aggregation, assembly and orientation

While the chiroptical activity of isolated molecular systems in dilute solution is often very weak, interactions between independent structures can enhance the *g*-factor. Here we present two examples with different origins: chromophore coupling and structural chirality.

### Intrinsic chiroptical activity: chromophore coupling

**Fig. 8:**
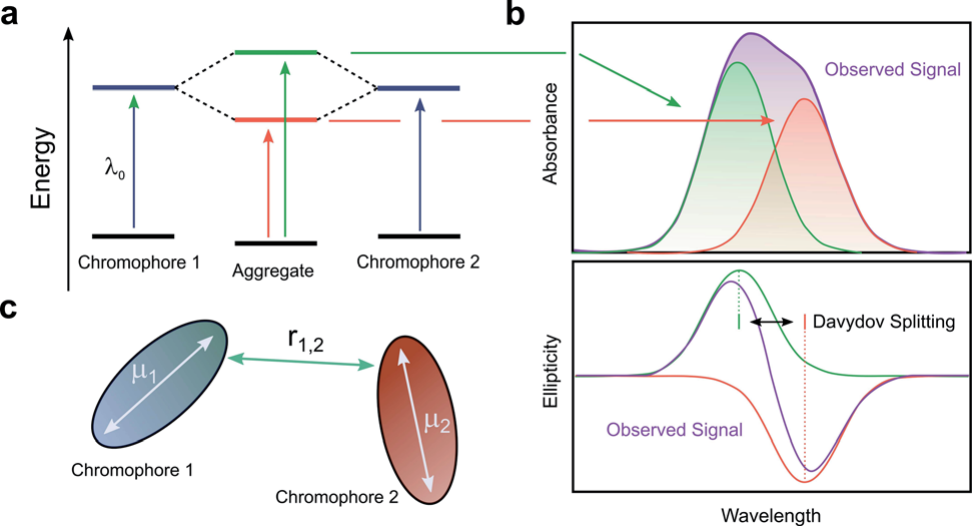
(a) The split excited states of two degenerate exciton-coupled chromophores. (b) The excitonic components (green, red) to the absorption (top) and CD (bottom) spectra and their overall observable spectra (purple). The CD spectra reveals the bisignate couplet (often referred to as a Cotton effect) typical of such coupling.^55^ (c) Schematic diagram of two chromophores highlighting the key quantities that determine the rotational strength *R*. The terms ***μ***_1_, ***μ***_2_ and *r*_12_ describe the two electric transition dipoles and their mutual distance.

When two or more chromophores are close to one another, interactions between their transition dipoles can result in coupling of the chromophores, giving rise to intense chiroptical phenomena.^50^ The relative orientation of the coupled chromophores and their transition dipoles may also introduce additional asymmetry to the system.^51–54^ Whilst various combinations of transition dipole interactions can occur, the most significant case arises when two nearby electric dipole allowed transitions couple to each other (so-called exciton coupling). The coupling of two oscillating dipoles causes the excited state to split into two non-degenerate levels (Fig. 8, with the energy difference being commonly referred to as the Davydov splitting) of high and low energy, depending on whether the dipoles couple in- or out-of-phase.^55^ If the coupled transition dipole moments are not co-planar, the ***m*** at the end of one oscillating dipole will be non-orthogonal to the other. This will result in a characteristic bisignate couplet appearing in the CD spectra, with rotational strengths given by *R*:^55,56^

4 *R* ∝ ±*r*_1,2_·***μ***_1_ × ***μ***_2_

Here, ***μ***_1_, ***μ***_2_ and *r*_1,2_ describe the two electric transition dipoles and their mutual distance.

The intensity of the chiroptical response, which can be evaluated by considering both *R* and the Coulomb potential of the interactions of ***μ***_1_ and ***μ***_2_, is directly proportional to the fourth power of the dipole strength (|***μ***_1,2_|), and inversely proportional to the square of the distance (*r*_1,2_) between the coupled chromophores.^55,56^ It is important to note that eqn (4) assumes that the intrinsic magnetic dipole moments of the coupled chromophores (***m***_1_ and ***m***_2_) are negligible, such that ***μ***–***μ*** coupling dominates the chiroptical response. If that is not the case, eqn (4) can be adapted to include terms relating to the coupling of ***μ*** and ***m***;^57^

5 *R* ∝ ±*r*_1,2_·***μ***_1_ × ***μ***_2_ + Im{(***μ***_1_ ∓ ***μ***_2_)·(***m***_1_ ∓ ***m***_2_)}

Excitonic coupling can manifest over a variety of structure types, including, (i) discrete small molecules; (ii) longer helically-folded macromolecules; (iii) assemblies of discrete molecules held together by supramolecular interactions or in the condensed state.

The extent to which exciton coupling between small discrete chiral molecules can enhance the chiroptical response has been investigated by a number of groups.^58–60^ Aromatic diimide and phenylenevinylene motifs are commonly employed in these studies due to their tendency to aggregate into well-defined structures in solution.^59,61,62^ For example, Nakashima, Kawai and co-workers investigated the chiroptical properties of two perylene bisimide (PBI) units tethered to a chiral binapthyl core.^60^ The CD spectra exhibited a clear bisignate CD band (*λ* = 540 nm), that is indicative of (intramolecular) excitonic coupling between the PBI units, achieving a |*g*_lum_| of 3 × 10^−3^. This dissymmetry was enhanced when the individual monomeric units were further assembled into spherical aggregates (26 nm in size), which combine both intra and intermolecular exciton coupling, achieving a |*g*_lum_| of 1.5 × 10^−2^ at 630 nm.^29,51,60^

**Fig. 9:**
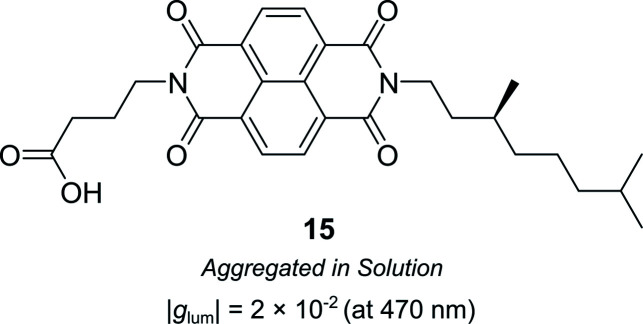
Structure of a chiral naphthalene diimide displaying point chirality that assembles into chiral aggregates in solution.^63^

Supramolecular chiral assemblies can enhance excitonic coupling, increasing the size of the active chiral component and the associated dissymmetry. For example, Meijer, Di Bari and co-workers studied the aggregation of a chiral naphthalene diimide (NDI, **15**) into supramolecular structures (Fig. 9).^63^ Whilst the monomers themselves exhibit point chirality, the stereogenic centre is sufficiently removed from the chromophore such that only the large-scale helically aggregated state affords any chiroptical response. The close positioning of the helically stacked NDI chromophores facilitates excitonic coupling, evidenced by the bisignate line shapes in CD spectra, which resulted in a |*g*_lum_| of 2 × 10^−2^ at 470 nm.^63^

**Fig. 10:**
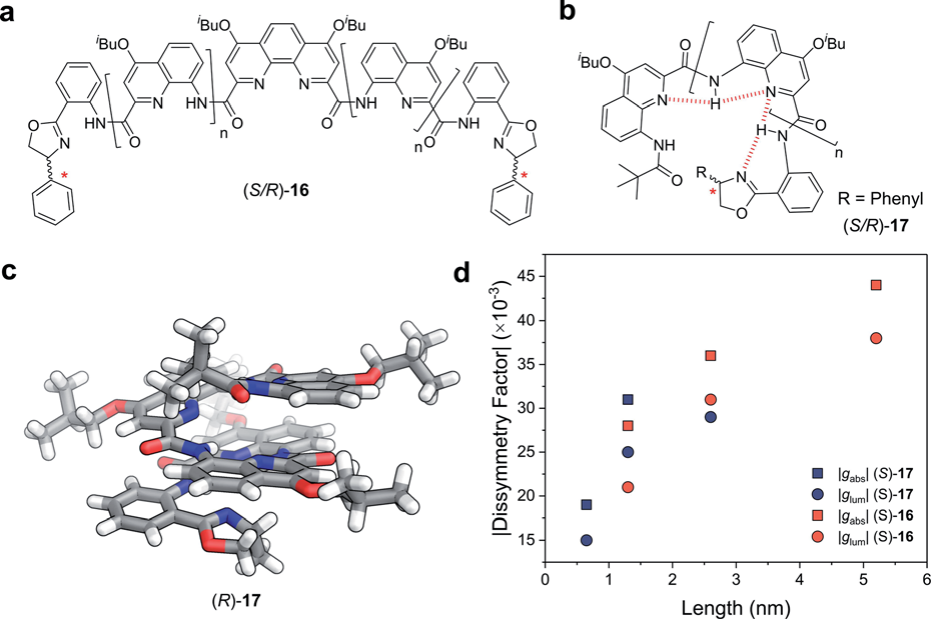
Panes (a and b) display examples of chemical structures that give rise to foldamers. The judiciously placed hydrogen bonds dictating the folding of the molecule are highlighted in red in pane (b). (c) Single crystal X-ray structure of an analogue of the foldamer shown in pane (b). (d) The |*g*_abs_| (*ca.* 388 nm) and |*g*_lum_| (*ca.* 430 nm) of the foldamers shown in pane (a and b). The lengths were calculated based on the size of a 4-repeat unit of CQ_4_.^66^

Covalent extension of the molecular framework can also give rise to large chiroptical activity through exciton coupling. A relevant example can be found in the helically folded oligomers called foldamers.^64^ Aromatic oligoamide foldamers make use of amide-coupled polyaromatic ring systems to achieve a robust helical structure, reaching lengths in excess of 7 nm.^65^ However, only recently have their chiroptical properties been studied quantitatively.^66,67^ Of these recent investigations, Jiang and co-workers designed a series of foldamers, **16** and **17**, that achieved |*g*_abs_| and |*g*_lum_| values as high as 4 × 10^−2^ (Fig. 10). The *g*-factors depend on the foldamer length and the structural rigidity of the helix, with longer foldamers achieving a more rigid structure and a higher *g*-factor.^66^

**Fig. 11:**
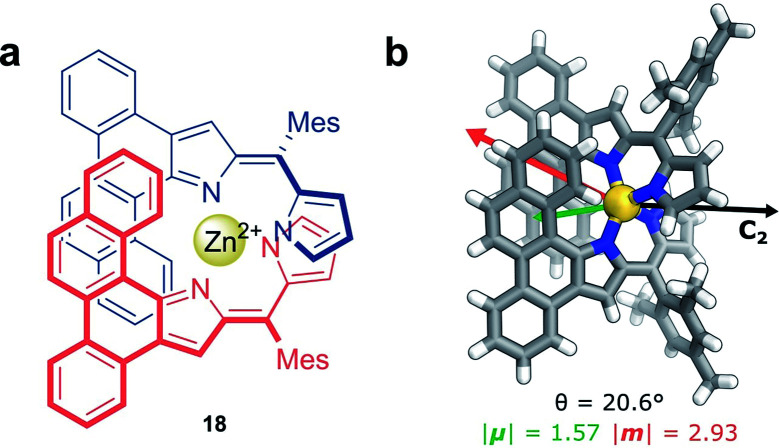
(a) Achiral ligands assemble around a Zn(ii) centre affording homoleptic helicate **18** that displays a C_2_ rotational axis. (b) The arrangement of the ligand facilitates exciton coupling.^68^

Non-covalent interactions, such as metal–ligand coordination, can also be employed to precisely orient chromophores for efficient exciton coupling. For example, Hasobe and co-workers have reported relatively large dissymmetry factors, |*g*_lum_| of 2 × 10^−2^, for a Zn(ii) helicate (**18**, Fig. 11a).^68^ The magnitude of the dissymmetry was attributed to the dimeric arrangement of the ligands around the metal ion, serving to promote exciton coupling between the ligands, as well as improve the alignment of |***μ***| and |***m***| (Fig. 11b).

**Fig. 12:**
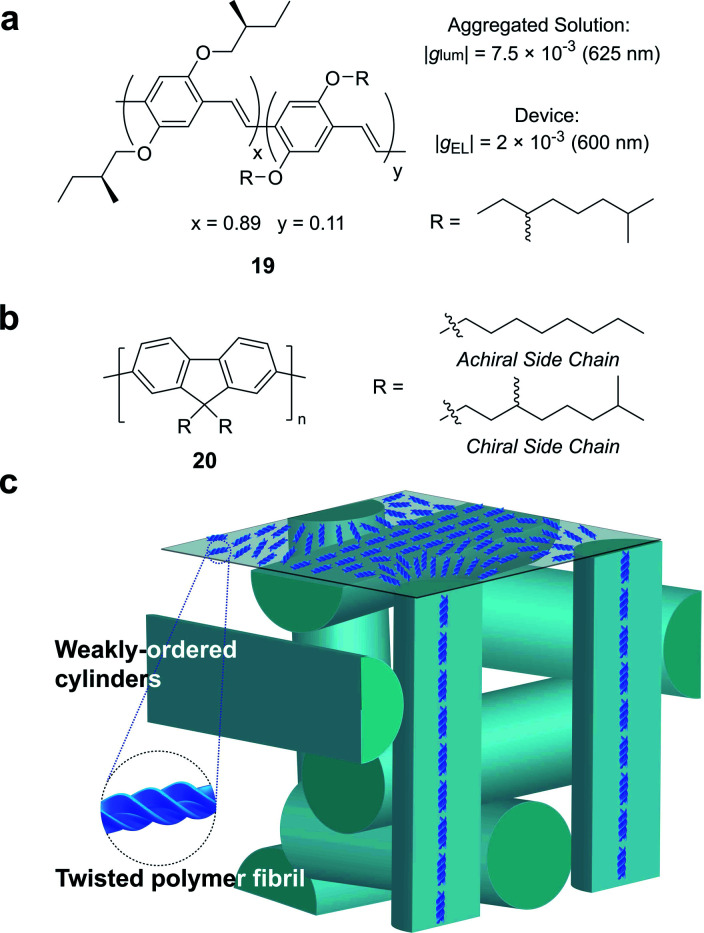
(a) Structure of a poly(*p*-phenylene vinylene) (PPV) polymer used to achieve CP electroluminescence (EL).^69^ (b) Structure of achiral and chiral polyfluorene (PF) polymers. (c) A representation of the proposed double twist cylinder blue phase.^31^

Partly driven by their high utility as organic electronic materials, the chiroptical activity of conjugated polymers has proved a rich area of study. In 1997, Meijer and co-workers published the first account of CP electroluminescence (EL) from chiral sidechain poly(*p*-phenylene vinylene) (PPV)-based devices (**19**, Fig. 12a, |*g*_EL_| 2 × 10^−3^ at 600 nm).^69^ PPV typically forms glassy phases in thin films, with little long-range order or any significant chiroptical response.^70^ The authors revealed that the |*g*_abs_| and |*g*_lum_| could be improved by a factor of 2 through the optimisation of interchain order (*i.e.* formation of chiral aggregates in solution *via* slow cooling or annealing of thin films) which enhances exciton coupling.^70^

While this seminal study showed the potential for chiroptical responses in condensed polymer phases, much larger chiroptical activity has been shown for thin films of polyfluorene (PF), **20**, and its co-polymer derivatives (Fig. 12b).^71–75^ Two of the most common approaches to induce chiral phases in thin films of such polymers are the introduction of chiral sidechains,^70,76,77^ and blending achiral polymers with chiral small molecule additives.^78–80^ The impact of the morphology,^81^ molecular weight^70^ and sidechain length^82^ on the chiroptical properties of thin films of PF derivatives have been extensively studied and *g*-factors >0.2 are routinely achievable. We recently undertook a mechanistic study to elucidate the origin of such large chiroptical activity, induced through either chiral sidechains or chiral additives.^31^ In the absence of liquid crystalline (LC) alignment layers (see below), we found that these systems exhibit very large intrinsic chiroptical activity (|*g*_abs_| ∼1). In contrast to previous assumptions, our structural data of thin films supports the assembly of twisted polymer fibrils into a weakly ordered double twist cylinder blue phase (Fig. 12c). The precise origins of such large intrinsic effects within such a structure remain to be fully defined, but the extension of the excited state over multiple chromophores (delocalisation), and exciton coupling between nearby polymer chains are key to describing the effects observed.

**Fig. 13:**
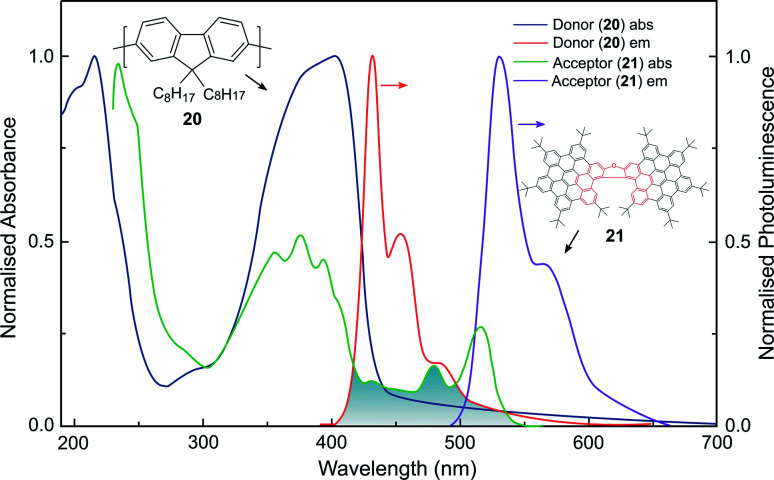
The normalised absorption and photoluminescence (*λ*_ex_: 385 nm) spectra of thin films of the donor (**20**, thickness, *t* = 140 nm) and acceptor (**21**, *t* = 90 nm) systems used to achieve FRET. The overlap between the donor emission and acceptor absorption is shaded. This figure has been generated from the data present in the ref. 84.

Whilst the discussion thus far has focused on the coupling of electric dipole allowed transitions (***μ***–***μ***), the interplay between the ***μ***–***μ*** and ***μ***–***m*** coupling of a donor–acceptor system is also of relevance in energy transfer mechanisms involving chiral species.^52,83^ For example, we recently demonstrated a 500-fold amplification of the |*g*_lum_| of a π-extended superhelicene, **21**, when embedded in an achiral conjugated polymer matrix.^84^ We proposed that the amplification arises through electrodynamic coupling between the electric and magnetic transition dipoles of the polymer donor and superhelicene acceptor, resulting in CP fluorescence resonance energy transfer (Fig. 13). It remains to be seen whether this approach can be more broadly adopted to enhance the *g*-factors of small organic molecules.^84^

### Non-intrinsic chiroptical activity: structural chirality

**Fig. 14:**
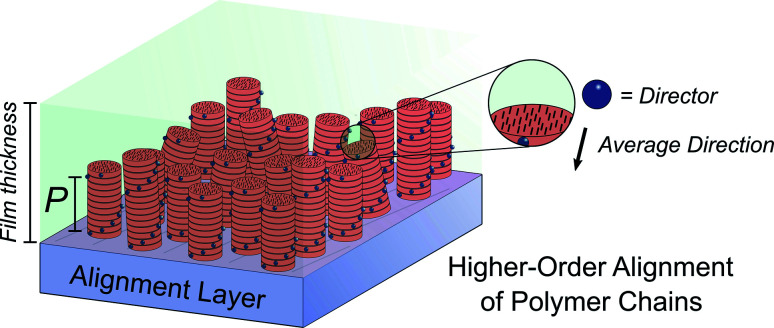
Schematic representation of a thin film containing polymers (black rods) arranged in cholesteric stacks (red columns) on an alignment layer. Blue spheres indicate the direction of the alignment of the polymeric chains. The helical pitch, *P*, corresponds to the length required to achieve a full helical turn.

Molecular ordering and higher-order assembly does not solely impact the intrinsic chiroptical response. When the helical pitch of chiral supramolecular assemblies' manifest over length scales corresponding to the wavelengths of their emission/absorption maxima, intense chiroptical phenomena can occur. Perhaps the most representative example is the so-called ‘cholesteric’ chiral nematic phase of LC materials.^85^ The cholesteric phase exhibits a helical supramolecular structure, whereby layers of rod-like molecules are twisted with respect to the layers below. Typically, the axis of helical organisation is perpendicular to the plane of the substrate, and the ordering is achieved using LC alignment layers and chiral twisting agents. Different to the intrinsic absorption or emission of CP light, these examples exhibit circular selective reflection/transmission (Bragg reflection). Cholesteric stacks reflect (and transmit) CP light with a wavelength (*λ*) that correlates to the pitch length (*P*) (eqn (6)): the distance along the helical axis that results in the rod-like molecules rotating 360° (Fig. 14).^86^

6 *λ* = *nP*

Here *n* is the average refractive index, calculated from the ordinary and extraordinary indices of refraction which are measured parallel and perpendicular to the uniaxial rod-like molecules.^87^

As light propagates through the helical supramolecular structure it becomes CP due to circular selective scattering and reflectance.^75^ As a result, the *g*-factor of light travelling through such materials is clearly dependent on the thickness of the chiral medium (and the number of pitch lengths).^88–90^ This mechanism of chiroptical activity is often referred to as structural chirality.^15^

Cholesteric LC materials have been covered extensively in the literature and have been utilised for a variety of technological applications, including reflective displays, augmented reality headsets, microcavity lasers and broad-band polarisers. There are many ways their structural chirality can be harnessed in CP light-dependent technologies and approaches.^91,92^ For example, it has been shown that the emitted light of achiral lumiphores embedded within chiral nematic (cholesteric) hosts can be significantly circularly polarised (*|g*_lum_| = ∼1.75 at 410 nm) after propagation through the chiral LC medium (thickness: 35 μm).^93^ This effect has more recently been harnessed in the amplification of CP emission from a large range of molecular emitters.^94^ Cholesteric LCs (thickness: 25 μm) have also been used in combination with electroluminescent polymer active layers to achieve CP output from an OLED by acting as a CP selective reflection/transmission filter (|*g*_lum_| = 1.57 at 560 nm).^95^

Alongside exciton coupling, structural chirality can be used to amplify the chiroptical activity of conjugated polymers. As highlighted above, PF and its co-polymers are well known to give rise to large chiroptical effects in thin films, often attributed to the formation of a (multi-domain) cholesteric phase.^77,82,96^ The molecular packing of such polymers is influenced by the presence of an LC alignment layer, such as unidirectionally rubbed polyimide. In the presence of an LC alignment layer, thermally annealed chiral thin films of polyfluorene-based polymers can form a cholesteric stack-like structure, where CP selective reflection/transmission dominates the chiroptical response. As discussed above, in the absence of the alignment layer, the coupling of electric and magnetic dipoles dominates the chiroptical response.^31^

The combination of liquid crystalline emissive materials and precisely controlled alignment layers can entirely circumvent the need for chiral materials entirely in CP devices. For example, Kim, Yu and co-workers have shown that structural chirality can be induced in achiral copolymer (F8BT) CP OLEDs through the use of carefully oriented LC alignment both sides of the active layer.^97^ After thermal annealing, the LC alignment layer serves to both template backbone orientation and as a hole transporter. Such films exhibit small CD (|*g*_abs_| ∼10^−3^), but can achieve |*g*_lum_| >0.5.

In summary, the chiroptical activity of molecules can be dramatically enhanced through aggregation, assembly and in the condensed phase. Indeed, such approaches are known to give some of the largest reported *g*-factors (>0.2) outside of the lanthanide molecular emitters. These high dissymmetry factors tend to occur in large (*i.e.* polymeric) molecular systems. The precise origin of such large dissymmetry factors can be very different, broadly grouped into intrinsic chiroptical activity *versus* structural chirality. It is therefore important to consider which mechanism(s) is better suited for a given approach/application. For example, structural chirality is a useful way to engineer a CP output from an emitter, which can even be achiral, and potentially allows the optimisation of the emitter (wavelength, quantum yield, *etc.*) to be decoupled from the optimisation of dissymmetry. On the other hand, for applications that require thin (∼100 nm) films, such as organic electronic devices (see below), structural chirality generally requires films that are too thick (>300 nm) to achieve efficient devices. Therefore, large intrinsic chiroptical activity is more favourable in such scenarios.^31^

## Enhancing chiroptical activity through modulation of the light

**Fig. 15:**
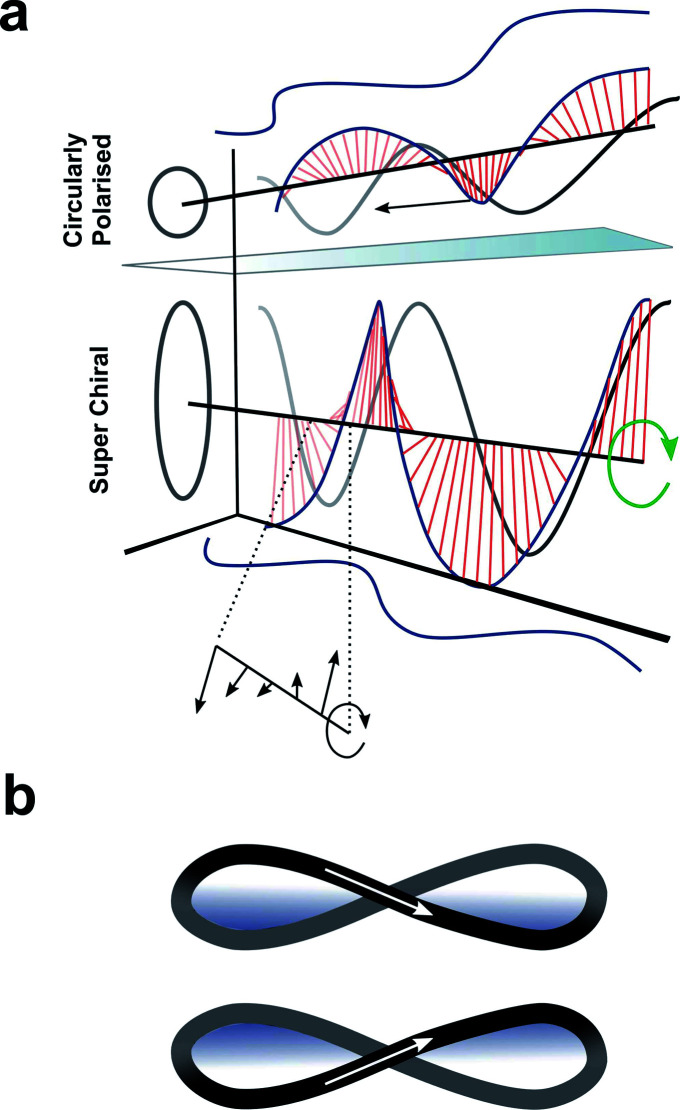
(a) Figure showing differences between CPL and SCL as proposed by Tang and Cohen.^14^ Electric field of left-handed CP light along with projections of the field onto the *xy*, *xz*, and *yz* planes. The arrows indicates direction of propagation of the field. (b) The left- and right-handed Lissajous curve reported by Ayuso and co-workers.^101^

Up to this point we have focused exclusively on the matter part of the light–matter interaction; exploiting changes in molecular structure and controlling higher-order assembly to improve the chiroptical response. An alternative approach would be to modulate the light to engineer a better chiroptical response. One such strategy towards this end is to exploit so-called superchiral light (SCL), although the use of this term is hotly debated.^98^ Tang and Cohen describe SCL as the combination of two counter-propagating CPL plane waves of opposite handedness, with equal frequencies but different intensities, generating an optical standing wave field (Fig. 15a).^14^ Within this standing wave the electric field vectors rotate nearly 180° in a distance much shorter than half the free-space wavelength, ideally over molecular dimensions (Fig. 15a, bottom). From this description, it was proposed that the dissymmetric interaction of light with chiral molecules can be enhanced by placing the molecules in the vicinity of one of the SCL nodes of the standing wave (such that *g*_SCL_ > *g*_CPL_). On the other hand, Coles and Andrews have proposed that these amplified dissymmetric interactions are not in fact due to nodal enhancements, but simply the result of beam superposition.^98,99^

As described in the introduction, CP light has long been studied in enantioselective synthesis, but the asymmetric induction is usually very low (<1% ee) due to low |*g*_abs_|. By using SCL, it should be possible to obtain enhanced enantioselectivity through improved dissymmetry for small molecules.^14^ There are currently limited examples of this approach, particularly due to the complicated and non-scalable setups required. Nonetheless, Tang and Cohen have shown an 11-fold enhancement over CPL in discrimination of the enantiomers of a PBI derivative by SCL and Zhang, Zou and co-workers have used SCL to impart a greater chiral bias in the asymmetric photo-polymerisation of diacetylene (achieving a 4-fold enhancement over CPL).^14,100^

Andrews and Forbes have shown that it is possible to enhance chiroptical responses through manipulation of the orbital angular momentum (OAM) delivered by structured beams of light.^102–104^ For example, the generation of surface plasmon optical vortices on chiral metasurfaces can elicit a stronger dissymmetric response from any molecules adsorbed on to such structure than one would typically expect. Recently, Ayuso and co-workers proposed a new form of synthetic chiral light,^101^ in which the electric field traces out a three-dimensional Lissajous curve in time at every fixed point in space (Fig. 15b). Compared to conventional CPL, Ayuso's synthetic chiral light does not rely on the helical propagation of the electric field. Instead, it is *locally* chiral, and, unlike CPL, remains so in the dipole approximation. As the interaction of chiral light with chiral matter is enantioselective in the dipole approximation (*i.e.* on a length scale of small molecules), synthetic chiral light should enable giant dissymmetric responses.

Beyond SCL, OAM and other structured forms of light, an alternative means to amplify weak chiroptical signals is to make use of non-linear phenomena. Nonlinear optical phenomena can be observed when the optical field strength of the excitation beam is comparable to the fields which bind valence electrons in a molecule. In these effects ***μ*** and ***m*** can be closer in magnitude than they are in linear optical measurements, which can give rise to much higher *g*-factors (eqn (3)). In 2019, Valev and co-workers used silver nanohelices in the first report of optical activity in non-linear Hyper–Rayleigh scattering (HRS), a technique where two photons interact with a non-centrosymmetric molecule to create a single photon with twice the incoming frequency.^1^ Verreault, Olivier, Rodriguez and co-workers first demonstrated the same effect in a molecular system in 2020.^105^ Application of HRS to a highly polarisable hexamer of 8-amino-2-quinolinecarboxylic acid led to a non-linear optical response of −0.39 when reported in an equivalent way as the *g*-factor (*cf.*eqn (1)).

The manipulation of light and optical processes can be exploited in such a way to enhance the dissymmetry observed for molecules which exhibit low *g*-factors for CP light. Such behaviour holds significant promise in the development of new spectroscopic methods to interrogate chiral substances that exhibit a low dissymmetry.^1–4^ Whether such approaches have broader impact in applications exploiting light–matter interactions remains to be seen.

## Outlook

In this perspective we explore the different factors that can be employed to increase the dissymmetry of the absorption/emission of CP light by chiral substances. As supported by the discussion above, it is clear that the mismatch between the ‘size’ of light and the size of the molecular system plays a fundamentally important role in the chiroptical response/dissymmetry factor of molecules. As is often the case however, the devil is in the detail. There are exceptions to the ‘molecular’ *g*-factor limit of small molecules (*i.e.* ∼10^−2^), best exemplified by chiral lanthanide complexes. There are also examples of relatively large supramolecular arrays where the *g*-factor is no better than small chiral molecules. A clear dependence on size is seen for systems that demonstrate structural chirality since CP-selective reflection or transmission is directly linked to the size (thickness) of the structure itself. However, for systems with intrinsic chiroptical activity, *g*-factor improvements through larger chromophores generally suffers from diminishing returns, particularly when the size of the chromophore approaches the effective conjugation length. Ultimately, it is difficult to draw (at least quantitative) comparisons between structurally distinct classes of molecules, given the contribution of molecular design, the optical transitions of interest, and assembly/alignment. Nonetheless, in this perspective we have attempted to present some of the key underlying principles that govern the magnitude of the chiroptical response beyond simple size considerations.

Of course, the strategy taken to control the level of dissymmetry in the chiral light–matter interaction very much depends on the area of study/application of interest. To conclude, we present three examples to give further context to such considerations:

### (1). Chiroptical organic electronic devices

These devices seek to utilise chirality in the organic material to detect or generate CP light for photonic applications. Such devices include organic light-emitting diodes (OLEDs), organic photodiodes (OPDs) and organic (photo) field-effect transistors (OFETs). The precise workings of the devices, state of the art materials and potential applications are beyond the scope of this perspective, but readers are directed to excellent reviews on the topic.^106,107^ In all cases, a high dissymmetry factor (>0.1), paired with excellent electrooptical performance, is required from these devices to be technologically relevant to ‘real-world’ applications. Taking OLEDs as a representative example, there are now excellent approaches to design high efficiency small molecule emitters for OLEDs. These include phosphorescent metal complexes and all-organic molecules that exhibit thermally activated delayed fluorescence (TADF).^108,109^ While efforts have been taken to make chiral versions of such small molecules, in almost all cases |*g*_lum_| is impractically low (<10^−2^).^110^ In contrast, polymeric thin film systems can allow for the generation of CP-OLEDs with |*g*_lum_| >1. Furthermore, by focusing on polymeric systems that exhibit high intrinsic chiroptical activity, rather than structural chirality, it is possible to maintain the optimum thin film thickness (∼100 nm) required for high performance devices.^31^ Therefore, in our opinion, polymeric systems appear to hold the most promise for applications that rely on large dissymmetry factors from thin film materials.

### (2). Chiral molecules for use in imaging applications

Using an emissive chiral molecule as a ‘tag’ for imaging applications—for example as a readout for a biological assay—allows one to use chiroptical spectroscopy as the detection strategy. In principle, high dissymmetry should not be required for such an approach, especially when using chromophores which emit at a wavelength beyond the main absorption/emission properties of biomolecules: as long as the chiroptical response is measurable it should give a suitable signal. At this point however, it is important to highlight practical challenges associated with very low *g*-factors.¶¶These problems can be exacerbated when moving from solution to the solid state. The chiral materials community have proposed several experimental precautions that must be taken when characterising chiral systems. This includes protocols to ensure that there are no linear artefacts in chiroptical spectra, ways to mitigate for CP-selective scattering, means to disentangle intrinsic from aggregated CPL features, and methods to account for reflection losses at interfaces.^7,8,10,118^ While there are now improved spectrometers to reliably measure low |*g*_abs_|, the measurement of low *g*_lum_ values remains nontrivial, particularly when fluorescence occurs close to the absorption onset. For advanced imaging applications, for example using CPL microscopy, such instrumentation is still under development and not routinely available.^111^ Given these challenges, emissive molecules with enhanced dissymmetry factors still provide a practical advantage for biological imaging reagents. Chiral lanthanide complexes hold much promise in this regard. Not only do such materials have large |*g*_lum_|, enabling more robust measurement of CPL, but they exhibit luminescence with a longer lifetime. This allows for time-gating, where the measurement of CPL can be delayed relative to the excitation pulse, further reducing any noise from the system.^112–114^

### (3). Chiral light for asymmetric synthesis

As any chemist who works on synthetic methodology knows, a broad substrate scope for a new method is key to its wider adoption: a new chiral catalyst, for example, will only be broadly adopted by the community if it is useful across diverse substrates of relevance to a range of sectors (natural products, pharmaceuticals, agrochemicals, *etc.*). Given this, many of the strategies reported in this perspective will not be of relevance to those who want to use chiral light to drive asymmetric reactions, due to the need to precisely control the molecular structure of the chromophore. In other words, it may be possible to design a very precise substrate to undergo asymmetric photochemistry using CPL with high(er) ee, but the substrate scope will likely be severely limited. Given this, it seems that adjusting the type of light employed has more promise for a broadly adoptable approach to asymmetric photochemical synthesis. Indeed, structured light provides a means to increase the chiroptical response of a molecular system without requiring precise tailoring of the structure.^14^ Therefore, this approach will likely be of higher utility in enantioselective synthesis using CP light,^100^ at least once the optical setups have been adapted for preparative chemistry and are more available to synthetic chemistry laboratories. Alternatively, chiral surfaces, containers and cages may provide another options to achieve higher selectivity in asymmetric synthesis.^115^ For example, chiral surfaces that may combine plasmonic effects with chiral recognition have recently been employed in enantioselective photosynthesis, affording ee's of up to 5%—comparable or better than those achieved with using CP light.^116^ This promising approach opens possibilities of using chirally patterned surfaces in heterogenous catalysis.

In summary, understanding and controlling chiral light–matter interactions remains of high academic importance for investigating and exploiting chiral materials. As design rules for the generation of molecular systems capable of achieving large *g*-factors continue to emerge, synthetic chemists will be able implement a more focused design of systems exhibiting a strong chiroptical response. This will undoubtedly lead to breakthroughs that pave the way for new CP-dependent applications across many sectors, including next-generation displays, encrypted optical communications, quantum computation and biosensing. Moreover, with access to materials displaying large *g*-factors, we anticipate the breadth of realised applications that benefit from chiral materials will continue to grow.

## Author contributions

M. J. F. conceived the ideas that underpin the perspective. J. L. G., J. W., J. R. B., T. J. P. and X. S. conducted the literature research and drafting of the manuscript. J. L. G. and J. W. designed the figures. All contributors were responsible for reviewing, editing and developing the perspective.

## Conflicts of interest

There are no conflicts to declare.
